# Response to “An overview of health workforce education and accreditation in Africa: implications for scaling-up capacity and quality”

**DOI:** 10.1186/s12960-022-00761-w

**Published:** 2023-01-26

**Authors:** Alex Berland, Helen Ewing, Kathleen Capone, Erica Frank, Miriam Chickering

**Affiliations:** 1 IUBAT – International University of Business, Agriculture and Technology, Dhaka, Bangladesh; 2 Nurses International, Minneapolis, USA; 3 SEED Global, Boston, USA; 4 NextGenU, Vancouver, Canada

**Keywords:** Open educational resources, Educator development, Health workforce education

## Letter to the Editor

In their seminal paper, *An overview of health workforce education and accreditation in Africa: implications for scaling-up capacity and quality* (*Hum Resour Health* *20,* 37, 2022), the authors call for comprehensive action across the continent to strengthen the standards for professional education. We believe their article identifies a global issue about nursing education, accreditation, and healthcare practice. Recognizing the impacts of international migration of health workers and based on our collective experience on several continents, we agree that this is a global issue.

The authors recommended, *Matching competencies with population needs, as well as increasing capacities for health worker production and … harmonization of curricula, education standards, accreditation and … promoting career progression and retention of tutors*. In our view, the supply of capable tutors is a critical constraint or “bottle-neck”. Many junior tutors have themselves received limited instruction with few opportunities to observe excellent practice and in their new teaching roles, supervisory supports are limited and their workloads are heavy.

There is a need for more diverse and flexible learning to be offered in low-resource countries, especially in healthcare education. Chio [1] reviewed effective practices in providing online education in low-resource settings. The findings indicated online learners readily achieved the learning outcomes compared to those in traditional face-to-face classrooms. Erlandsson et al. [2] evaluated a model for capacity building of midwifery educators through a blended, web-based program. This research illustrated how the use of technology in online learning increased midwife educators’ professional competencies and skills. As internet access and smartphones become more available globally, it is increasingly possible to find alternative ways of educating nurses in critically underserved areas.

Our goal at Nurses International, a not-for-profit organization, is to support nurse educators with Open Education Resources using available technology to build evidence-based nursing curricula with an emphasis on critical thinking. We produce competency-based educational content and tools for developing and accessing clinical judgment. Our comprehensive *Educator’s Guide* includes theory and practice topics included in standard Master of Nursing Education programs [5]. The curriculum content is based on South Asian regulators' syllabi and with Creative Commons rules, they are all freely available for adapting to local context. To date, the Nurses International resources have been downloaded in over 100 countries. The all-volunteer effort is slower than we believe it should be. Therefore, we welcome collaborators at https://nursesinternational.org/ who are committed to open education, such as NextGenU.org [3].

We thank Okoroafor et al. for sharing their findings on new solutions and opportunities for healthcare workforce education. In Florence Nightingale’s words, “…Never lose an opportunity of urging a practical beginning, however small, for it is wonderful how often in such matters the mustard-seed germinates and roots itself.” [4].

## Data Availability

Not applicable.
